# Thymol nanoemulsion exhibits potential antibacterial activity against bacterial pustule disease and growth promotory effect on soybean

**DOI:** 10.1038/s41598-018-24871-5

**Published:** 2018-04-27

**Authors:** Sarita Kumari, R. V. Kumaraswamy, Ram Chandra Choudhary, S. S. Sharma, Ajay Pal, Ramesh Raliya, Pratim Biswas, Vinod Saharan

**Affiliations:** 1 Department of Molecular Biology and Biotechnology, Rajasthan College of Agriculture, Maharana Pratap University of Agriculture and Technology, Udaipur, Rajasthan 313001 India; 2 Department of Plant Pathology, Rajasthan College of Agriculture, Maharana Pratap University of Agriculture and Technology, Udaipur, Rajasthan 313 001 India; 3 Department of Chemistry and Biochemistry, College of Basic Sciences and Humanities, Chaudhary Charan Singh Haryana Agricultural University, Hisar, Haryana 125004 India; 4 Department of Energy, Environmental and Chemical Engineering, Washington University in St. Louis, St. Louis, MO 63130 USA

## Abstract

An antibacterial and plant growth promoting nanoemulsion was formulated using thymol, an essential oil component of plant and *Quillaja* saponin, a glycoside surfactant of *Quillaja* tree. The emulsion was prepared by a sonication method. Fifty minutes of sonication delivered a long term stable thymol nanoemulsion which was characterized by dynamic light scattering (DLS), transmission electron microscopy (TEM), cryogenic-field emission scanning electron microscopy (Cryo-FESEM) and fourier transform infra-red (FTIR) spectroscopy. Creaming index, pH and dilution stability were also studied for deliberation of its practical applications. The nanoemulsion (0.01–0.06%, v/v) showed substantial *in vitro* growth inhibition of *Xanthomonas axonopodis* pv. *glycine* of soybean (6.7-0.0 log CFU/ml). In pot experiments, seed treatment and foliar application of the nanoemulsion (0.03–0.06%, v/v) significantly lowered the disease severity (DS) (33.3–3.3%) and increased percent efficacy of disease control (PEDC) (54.9–95.4%) of bacterial pustule in soybean caused by *X. axonopodis* pv. *glycine*. Subsequently, significant enhancements of plant growth were also recorded in plants treated with thymol nanoemulsion. This is the first report of a thymol based nanoemulsion obtained using *Quillaja* saponin as a surfactant. Our study claims that nano scale thymol could be a potential antimicrobial and plant growth promoting agent for agriculture.

## Introduction

In recent years, unrestrained use of synthetic agrochemicals in crop protection has raised serious concerns of environmental contamination and resistance enhancement in phytopathogenic microbes. To address these issues, development of bio-based/non-synthetic biocides for agriculture has become an important research direction. Thymol(2-isopropyl-5-methylphenol), a major essential oil component (EOC) of plants from *Lamiaceae* family, possesses the phenolic hydroxyl group which might have contributed to its antimicrobial activity^1^. Furthermore, thymol has been classified as a Generally Recognized as Safe molecule by the U.S Food and Drug Administration in its use as a food additive^2^. Therefore, application of thymol has been widely reported in the medical^3^, food^4–8^ and agricultural field^9–11^. Owing to its biodegradable, strong antimicrobial and antioxidant nature, thymol has been sought to be utilized in a much broader manner in the agricultural sector^11–14^. However, it has low water solubility which reduces its biological activity and limits its application through aqueous medium^15,16^. In addition, lipophilic bioactive compounds such as essential oils (EOs) and essential oil components (EOCs), e.g. thymol, are physically and chemically unstable in the presence of oxygen, light and temperature, which reduces their efficiency^17–22^. These problems might be overcome by preparation of thymol nanoemulsions^23^ by converting it into nano-droplets through entrapment in suitable surfactants^15,24^. Due to this encapsulation, thymol becomes physically and chemically stable in the aqueous medium^15,25,26^. From a biological stand-point, fine droplets of nanoemulsion could be efficiently absorbed through biological surfaces for efficient and wider biological activities. A range of synthetic surfactants have been used by many researchers for the preparation of nanoemulsions^15,27–31^. In recent times, researchers have prepared thymol based emulsions using synthetic and few natural surfactants and determined their antimicrobial activity^15,16,19,24,29,31–35^. But, use of synthetic surfactants increases the artificial content in the emulsions, and makes them unusable in the agriculture/food industry and furthermore have negative environmental impacts^35,36^. In view of this, there is an urgent need to search biological based surfactants for preparation of thymol nanoemulsions for their safe and effective use in agriculture. Recent findings have unfastened a promising natural surfactant called saponin, a non-ionic glycoside from the bark of *Quillaja saponaria Molina*^37,38^. But, so far, no report has revealed the use of saponin in preparation of thymol nanoemulsions and moreover, thymol based nanoemulsions have also not been evaluated in plants for antibacterial and plant growth promoting activity. This study reports a reproducible, rapid and easy method for preparation of stable and effective thymol nanoemulsions using saponin, a natural surfactant. The primary goal of the present study is to use saponin as a surfactant to prepare stable thymol nanoemulsion and to test its antibacterial activity against *Xanthomonas axonopodis* pv. *glycine* that causes bacterial pustule disease in soybean. Another ancillary goal of the study was to decipher its effect on plant growth of soybean.

## Results

### Physicochemical characterization of the nanoemulsion

**Figure 1:**
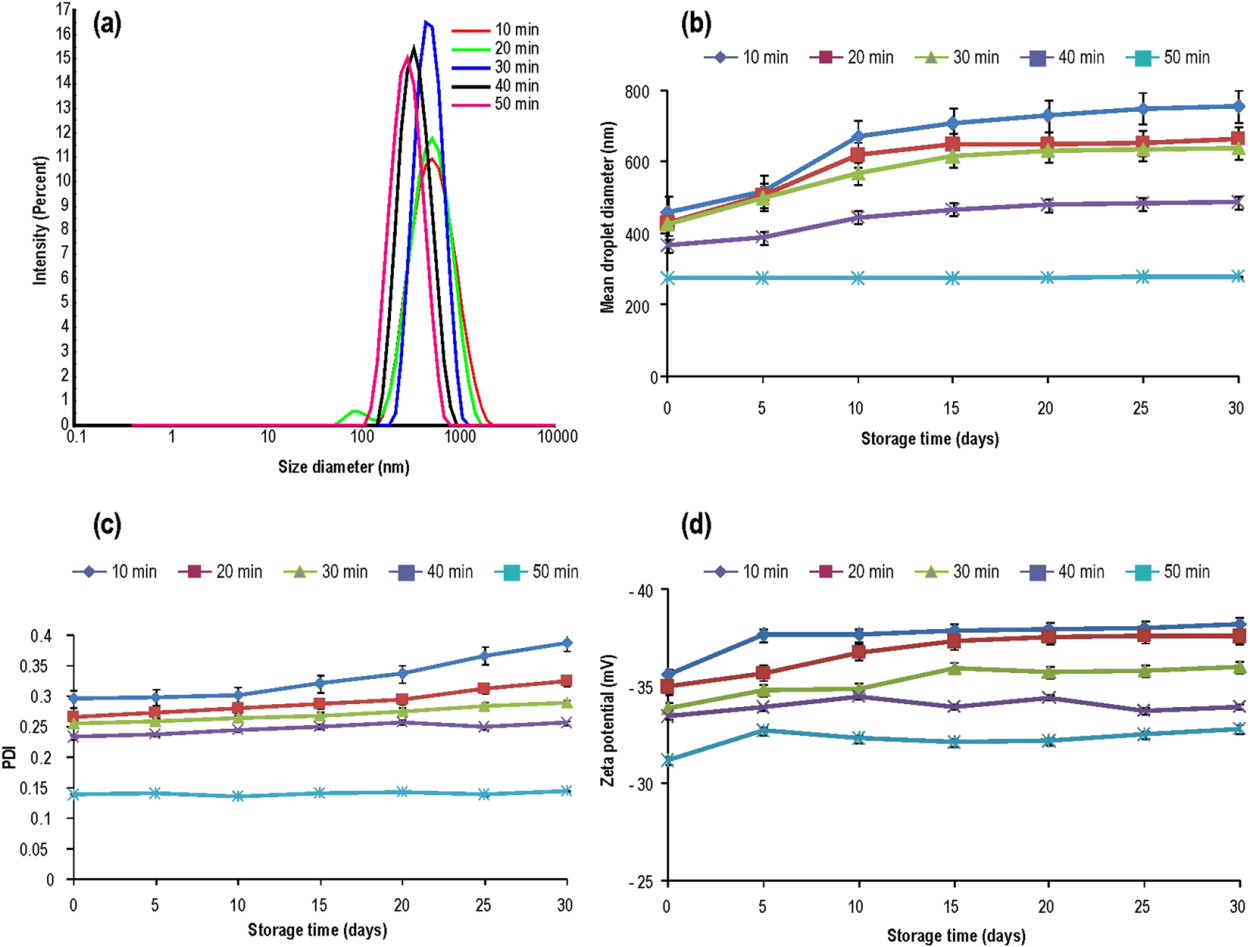
DLS analysis of nanoemulsions (**a**) size distribution of 10–50 min sonicated nanoemulsions at 0 day (**b**) a comparative graphical presentation of mean droplet diameter (Z-averages), (**c**) PDI and (**d**) zeta-potential of nanoemulsions (from 0 to 30 days) prepared by different sonication times. Error bars represent ± SE (standard error).

**Figure 2:**
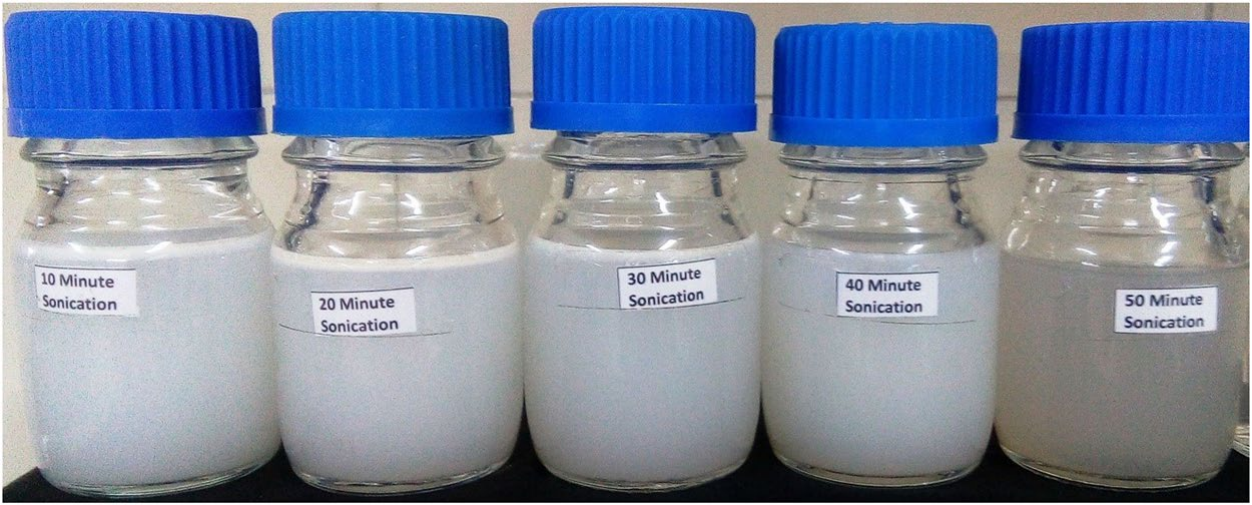
Photograph of thymol nanoemulsions prepared at different sonication time.

**Figure 3:**
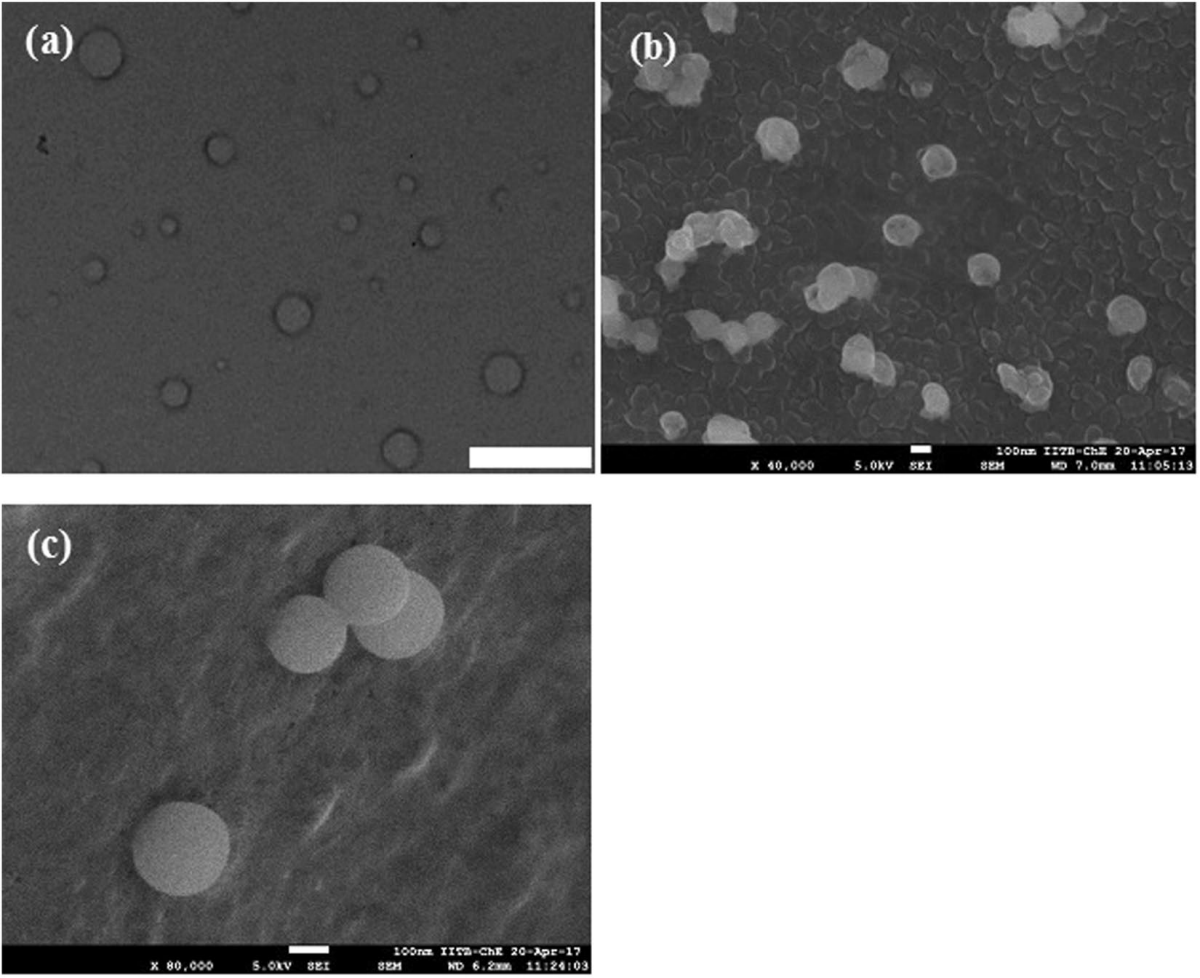
Morphological characterization of synthesized nanoemulsion**:** TEM image of (**a**) thymol nanoemulsion at 45kx, scale bar 500 nm. Cryo-FESEM images of (**b**) thymol nanoemulsion at 40 kx and (**c**) at 80 kx magnification.

**Figure 4:**
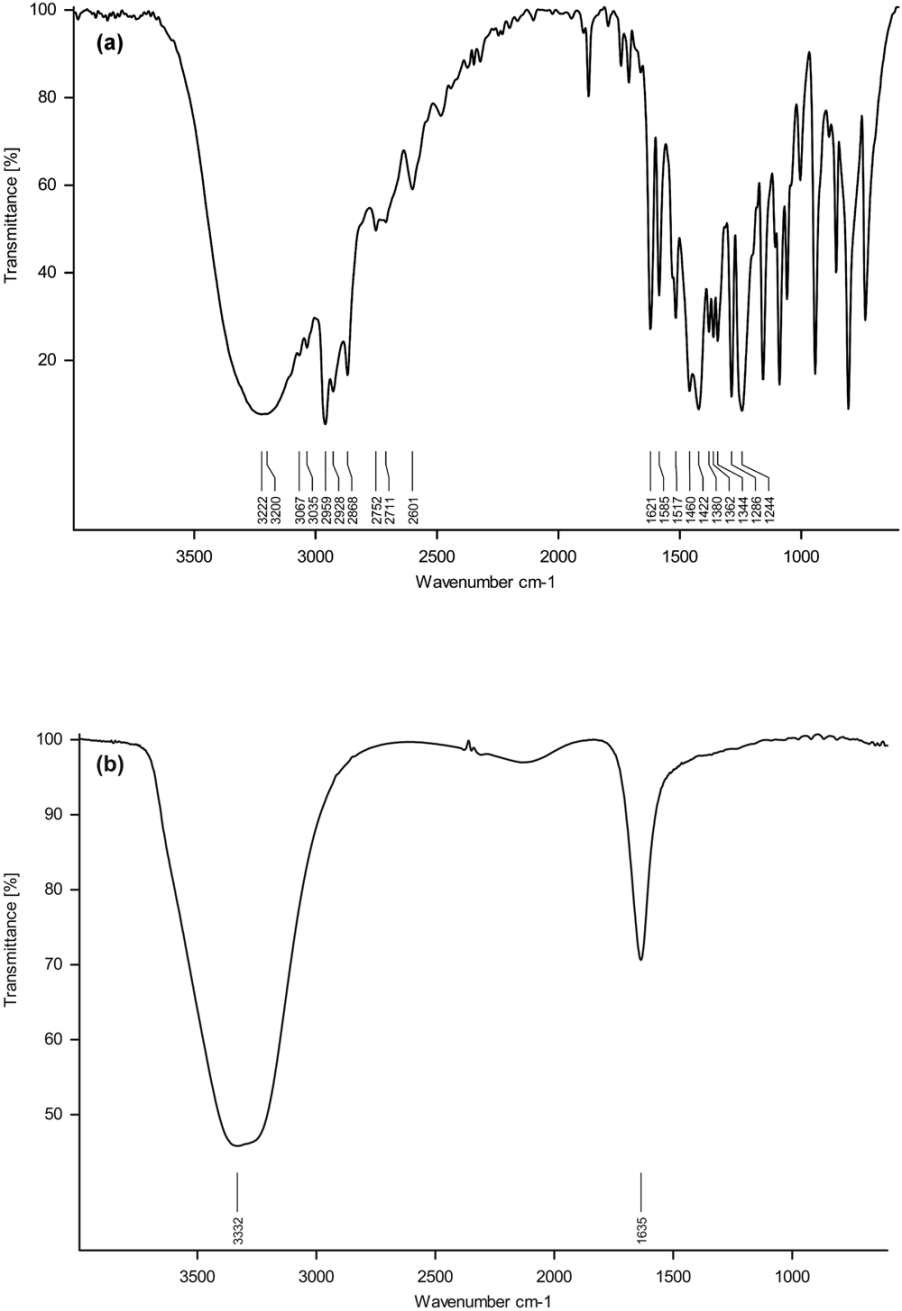
FTIR spectra of (**a**) bulk thymol and (**b**) thymol nanoemulsion.

Detailed characterization of nanoscale materials is essential to understand the phenomenon at the nano-biointerface. We have used dynamic light scattering (DLS), transmission electron microscopy (TEM), cryogenic-field emission scanning electron microscopy (Cryo-FESEM), fourier transform infra-red (FTIR) and stability study for the characterization of thymol based nanoemulsions. Sonication parameters used herewith were standardized for 100 ml reaction volume (containing thymol, saponin and water) in 250 ml glass beaker (9.5 cm height, 7.0 cm diameter). Sonication was carried out by placing probe in center of the 250 ml glass beaker, reaching 1.75 cm depth of 100 ml of reaction mixture. To manage the temperature below 35 °C during sonication, five second pulse rate (on/off) was found effective. In 8–10 min of sonication, reaction mixture turned milky and up to 50 min of sonication, a semi transparent dispersal appeared which indicates a nanoemulsion of stable physicochemical properties at room temperature. Freshly prepared nanoemulsions (day 0) showed a shift of size distribution from higher size to lower size range. Therefore a decrease in droplet diameter (z-averages) was recorded with increase in sonication time and the corresponding values were 458.5, 428.4, 425.4, 367 and 274.7 nm at 10, 20, 30, 40 and 50 min of sonication, respectively (Fig. 1a,b). Likewise, polydispersity index (PDI) values also decreased considerably (0.29, 0.26, 0.25, 0.23 and 0.13) with increasing sonication time (10, 20, 30, 40 and 50 min) (Fig. 1c). Zeta-potential of nanoemulsion faintly decreased with increase in sonication time (Fig. 1d). The stability of nanoemulsions at room temperature was also systematically investigated by measuring z-averages, PDI and zeta-potential up to 30 days at an interval of 5 days and finally after 3 months of storage. After 30 days, z-averages of nanoemulsions prepared by 10–40 min of sonication noticeably increased while it was unchanged in samples prepared with 50 min of sonication (Fig. 1b). After 30 days of storage, however, PDI and zeta-potential remained fairly invariably in nanoemulsions prepared by 40 and 50 min of sonication as compared to nanoemulsion obtained from 10 to 30 min of sonication (Fig. 1c,d). After 3 months of storage, 50 min sonicated nanoemulsion confirmed stability in terms of mean droplet size (293 ± 2.7 nm), PDI (0.15) and zeta-potential (−32 mV) as compared to other nanoemulsions (Table 1). Creaming was visually noticed after 20–30 days in nanoemulsion prepared by 10 to 40 min of sonication (Fig. 2). After 3 months of storage, 93.1, 94.8, 96.5, 99.6 and 100% creaming index (CI) was recorded in 10, 20, 30, 40 and 50 min sonicated nanoemulsions, respectively (Table 1). No phase separation (100% CI) was noticed in nanoemulsion obtained from fifty min of sonication (Fig. 2). At variable pH, mainly 3–7, nano-droplets of 50 min sonicated nanoemulsion remained quite stable with respect to size, PDI and zeta-potential (Table 2). The stability of the 50 min sonicated nanoemulsion was also determined after (500 and 1000-folds) dilution in deionized water. An increase in z-averages (962 ± 5 nm) and PDI value (0.84) was recorded in 1000-fold diluted nanoemulsion yet 70% of the droplets showed size below 100 nm (Fig. S1, Table S1). Moreover, 1000-fold diluted samples stayed homogeneous without any sign of creaming or precipitation. However, sonication above 50 min i.e. 60 min, shifted nano-droplets to bimodal size distribution (Fig. S2a) with PDI value reaching to 0.50 and 95% of droplets were below 100 nm (Fig. S2b; Table S2). Overall, nanoemulsion prepared by 50 min of sonication, showed non-significant changes in size, PDI and zeta-potential after 3 months of storage as compared to other nanoemulsions. TEM analysis showed spherical droplets of 80–150 nm in 50 min sonicated nanoemulsion (Fig. 3a). To further confirm the physical size and surface architecture of nanoemulsion in pristine, Cryo-FESEM analysis was carried out. Bright and smooth surfaced spherical nano-droplets of 90–180 nm were noticed (Fig. 3b,c). In FTIR analysis, nanoemulsion did not show any noticeable peak in spectrum. Although a smooth and widened peak was noticed at 3331 cm^−1^ which denotes the hydrophilic interaction (Fig. 4a,b). Thymol nanoemulsion synthesized by 50 min of sonication was further tested for antibacterial activity, disease control and plant growth promoting activity.

**Table 1:** Stability study of thymol nanoemulsion after 3 months.

Sonication time (min)	Z-average (nm)	PDI value	Zeta potential (mV)	% CI
10	783.83 ± 4.59^a^	0.383 ± 0.01^a^	−38.23 ± 0.63^c^	93.16 ± 0.08^d^
20	678.90 ± 5.84^b^	0.355 ± 0.01^ab^	−37.30 ± 0.65^c^	94.82 ± 0.12^c^
30	650.66 ± 3.18^c^	0.300 ± 0.04^ab^	−36.20 ± 0.50^bc^	96.55 ± 0.11^b^
40	498.73 ± 2.70^d^	0.252 ± 0.01^bc^	−34.06 ± 0.35^ab^	99.60 ± 0.02^a^
50	293.13 ± 0.93^e^	0.155 ± 0.01^c^	−32.66 ± 0.51^a^	100 ± 0.00^a^

Each value is mean of triplicates. Mean ± SE followed by same letter in column are not significantly different at p = 0.05 as determined by Tukey-Kramer HSD.

**Table 2:** Effect of pH on thymol nanoemulsion prepared by 50 min. sonication.

pH	Z-average (nm)	PDI value	Zeta potential (mV)
3	390.46 ± 9.46^c^	0.230 ± 0.02^a^	−12.97 ± 2.32^a^
4	300.16 ± 3.02^d^	0.226 ± 0.02^a^	−36.50 ± 3.85^bc^
5	280.50 ± 8.29^d^	0.181 ± 0.58^a^	−24.13 ± 1.20^ab^
6	381.26 ± 8.45^c^	0.275 ± 0.01^a^	−37.83 ± 4.69^bc^
7	393.13 ± 2.98^c^	0.246 ± 0.01^a^	−36.26 ± 1.95^c^
8	462.10 ± 9.35^b^	0.307 ± 0.09^a^	−45.23 ± 0.18^c^
9	606.30 ± 5.90^a^	0.331 ± 0.08^a^	−41.06 ± 1.12^c^

Each value is mean of triplicates. Mean ± SE followed by same letter in column are not significantly different at p = 0.05 as determined by Tukey-Kramer HSD.

### *In-vitro* antibacterial activity

**Figure 5:**
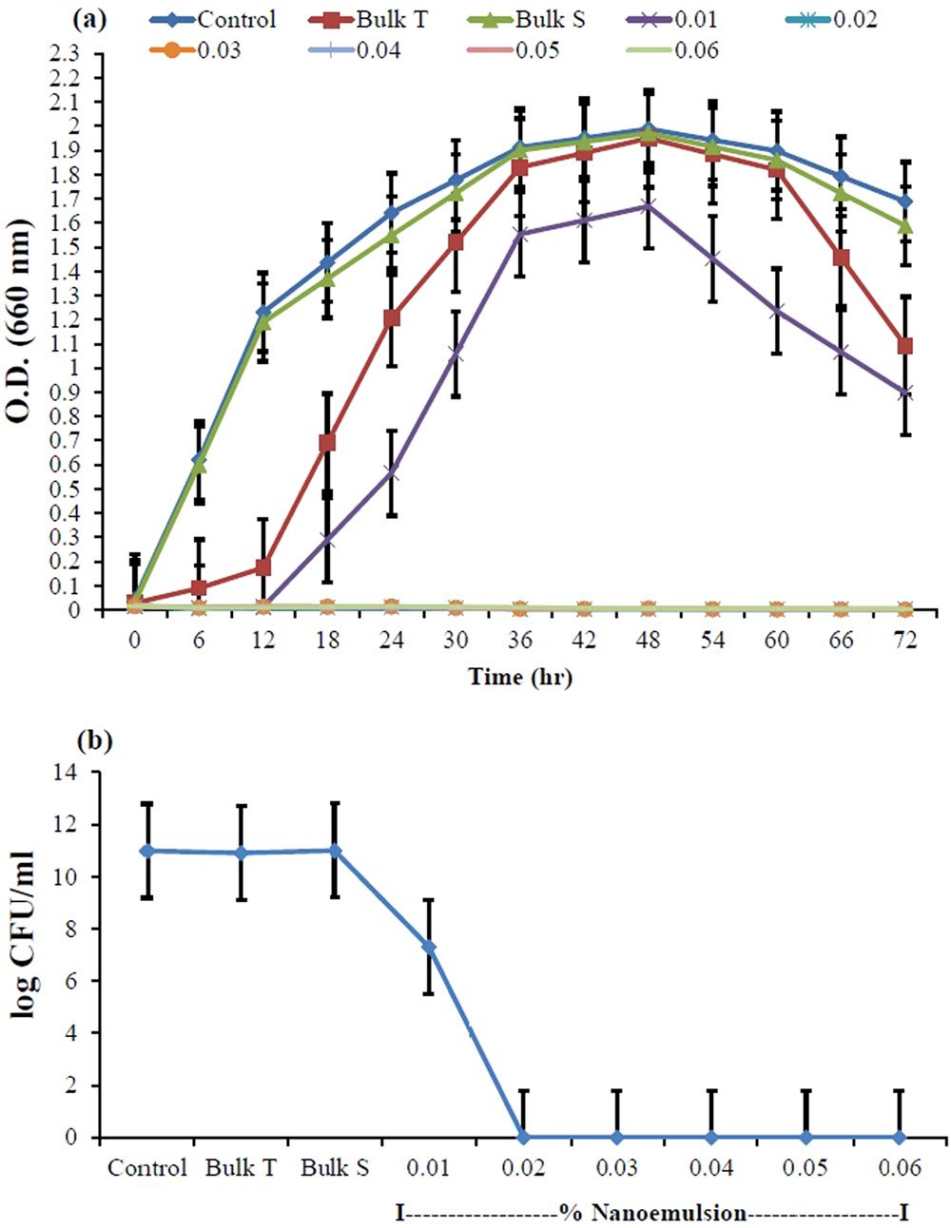
Antibacterial activity of control (water), bulk T (bulk thymol), bulk S (bulk saponin) and thymol nanoemulsions (0.01–0.06%, v/v) (**a**) as O.D and (**b**) CFU/ml. Error bars represent ± SE (standard error).

The growth kinetics studies of *X. axonopodis* pv*. glycine* in the presence of different concentrations of thymol nanoemulsion along with water (control), bulk thymol (0.01%, w/v) and bulk saponin (0.01%, w/v) are shown in Fig. 5. Time-kill curve showed a strong growth inhibitory effect by zero absorbance at 0.02 to 0.06% (v/v) concentrations of nanoemulsion (Fig. 5a). Further, antibacterial activity was measured in terms of log CFU/ml (Fig. 5b). Bacterial counts were almost the same in control (water), bulk thymol and bulk saponin, indicating the inefficacy of bulk thymol and bulk saponin on bacterial growth. Thymol nanoemulsion from 0.02 to 0.06% (v/v) concentrations completely inhibited bacterial growth by expressing 0.0 log CFU/ml (Fig. 5b). The observed data show a strong antimicrobial activity of thymol nanoemulsion against the bacterial strain under study.

### Effect of nanoemulsion on bacterial pustule disease and plant growth

**Figure 6:**
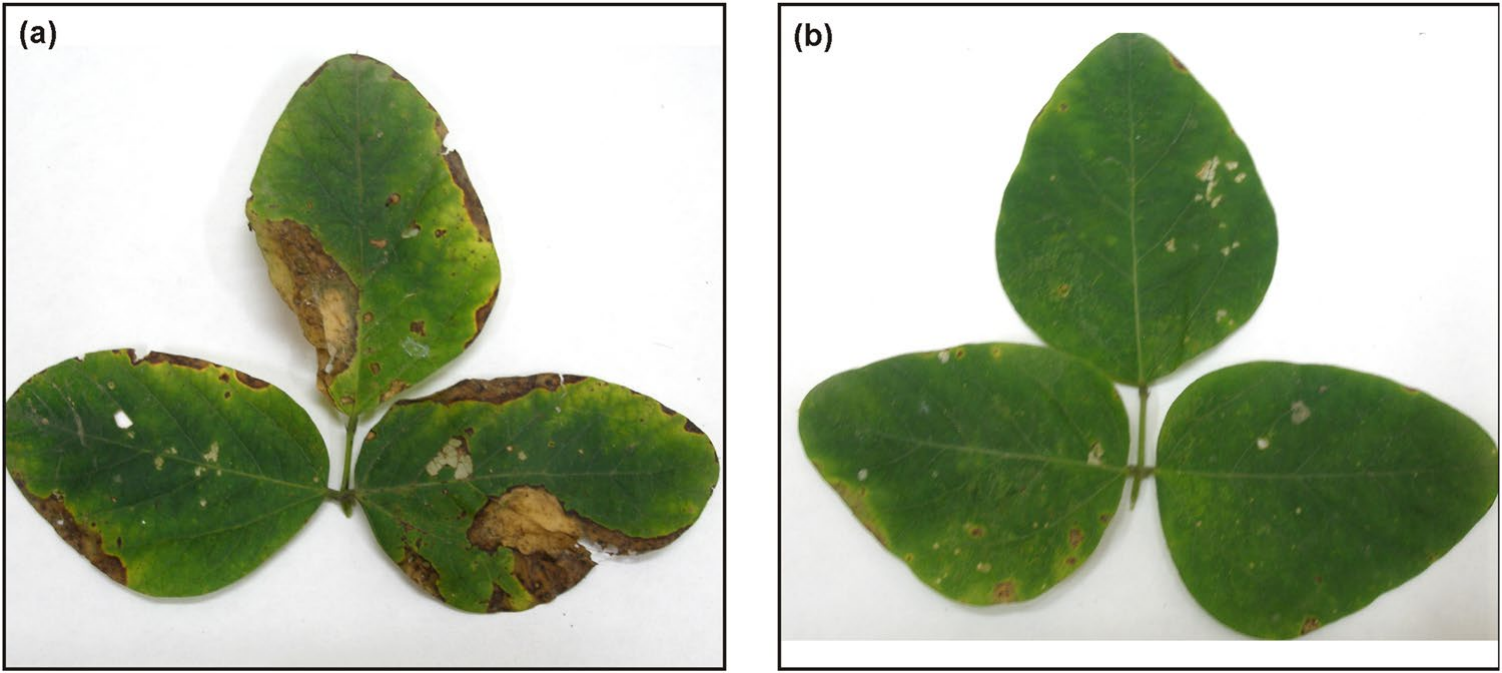
Symptoms of bacterial pustule disease on soybean plants in pot experiments (**a**) lesions expanded and merged in control (**b**) small yellow to brown lesions in soybean leaf at 0.06%,v/v thymol nanoemulsion.

**Figure 7:**
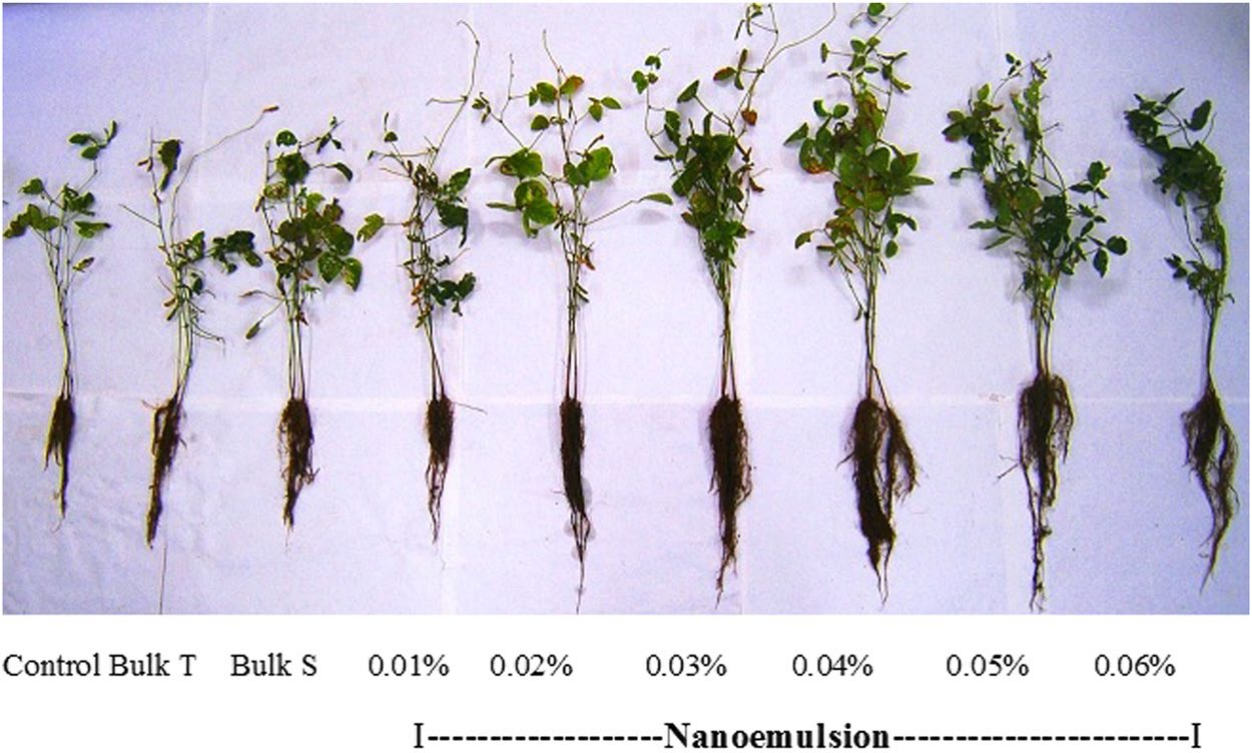
Effect of thymol nanoemulsion on plant growth of soybean. Concentrations of thymol nanoemulsions ranging from 0.02 to 0.06% v/v, exhibited visual differences in plant growth.

In batch experiments conducted in pots, bacterial pustule disease symptoms were observed after 10 days of artificial inoculation of *X. axonopodis* pv*. glycine*. Thereafter, foliar spray of water (control), bulk thymol, bulk saponin and different concentrations of nanoemulsion was applied. After 10 days of application, data for disease severity (DS) and percent efficacy of disease control (PEDC) were recorded. Small, pale-green spot with elevated pustule were critically analyzed on the experimental plants. In control plants, lesions expanded and merged leading DS to the extent of 74% (Table 3, Fig. 6a) while in thymol nanoemulsion treated plants, small yellow to brown lesions were observed (Fig. 6b). All plants treated with 0.01 to 0.06% thymol nanoemulsion showed significantly lower DS (59 to 3.33%). A maximum PEDC was recorded in plants sprayed with 0.06% thymol nanoemulsion (Table 3). Another aim of pot experiment was to ascertain the effect of thymol nanoemulsion on growth characteristics of soybean plant. Based on the preliminary experiments, 4 h seed treatment was effective in vigor seedling growth, therefore, before sowing; soybean seeds were treated with control (water), bulk thymol, bulk saponin and nanoemulsion as detailed in materials and methods. Thymol nanoemulsion treatments recorded significantly higher values of plant height, root length, root fresh weight, number of nodules/plant, weight/nodule, number of pods/plant and 100 seed weight as compared to control (water), bulk thymol and bulk saponin treatments (Table 4; Fig. 7). Although, at higher concentrations of nanoemulsion (0.06%), a slight decrease in various growth parameters were observed as compared to 0.02 to 0.05% of nanoemulsion treatments (Table 4). These findings divulge the growth promoting effect of thymol nanoemulsion on soybean plants.

**Table 3:** Effect of thymol nanoemulsion on bacterial pustule disease control in pot experiment of soybean.

Treatment (%)	DS (%)^A^	PEDC (%)^A^
Control^B^	74.00 ± 1.15^a^	0.00 ± 0.00^g^
Bulk S^C^ (0.01)	71.33 ± 1.76^ab^	3.62 ± 0.96^fg^
Bulk T^D^ (0.01)	68.00 ± 4.00^ab^	13.20 ± 1.37^ef^
**Thymol nanoemulsion**		
0.01	59.33 ± 4.66^bc^	23.06 ± 4.04^de^
0.02	54.00 ± 2.30^c^	27.03 ± 2.72^d^
0.03	33.33 ± 1.76^d^	54.95 ± 2.25^c^
0.04	29.33 ± 0.66^d^	60.34 ± 1.10^c^
0.05	16.66 ± 2.66^e^	77.36 ± 3.96^b^
0.06	3.33 ± 0.66^f^	95.49 ± 0.90^a^

^A^Each value is mean of triplicates and each replicate consisted of 3 plants samples. Mean ± SE followed by same letter in column of each treatment are not significantly different at p = 0.05 as determined by Tukey-Kramer HSD. ^B^Control with water. ^C^Bulk S (bulk saponin) dissolved in water and ^D^Bulk T (bulk thymol) dissolved in 1% DMSO (Dimethyl sulfoxide). DS (disease severity). PEDC (percent efficacy of disease control).

**Table 4:** Effect of thymol nanoemulsion on plant growth of soybean.

Treatment (%)	Plant growth^A^						100 seed weight (g)
	Plant height (cm)	Root length (cm)	Root fresh weight (g/plant)	Number of nodules/plant	Weight/nodule (mg)	Number of pods/plant	
Control^B^	53.16 ± 2.72 ^cd^	30.83 ± 0.72^ab^	0.79 ± 0.08^c^	7.75 ± 0.90^bc^	17.00 ± 2.1^c^	4.58 ± 0.44 ^cd^	4.96 ± 0.23 ^cd^
Bulk S^**C**^ (0.01)	53.58 ± 0.87 ^cd^	31.58 ± 2.55^ab^	0.93 ± 0.02^c^	5.83 ± 0.79^c^	21.33 ± 1.4^bc^	4.75 ± 0.38 ^cd^	4.36 ± 0.27^d^
Bulk T^D^ (0.01)	53.75 ± 2.91 ^cd^	31.75 ± 0.38^ab^	0.91 ± 0.06^c^	5.5 ± 0.50^c^	28.75 ± 2.1^ab^	5.58 ± 0.84^bcd^	4.63 ± 0.21 ^cd^
**Thymol nanoemulsion**							
0.01	54.5 ± 2.15^bcd^	31.83 ± 1.08^ab^	0.97 ± 0.06^c^	5.83 ± 0.44^c^	30.41 ± 0.65^ab^	5.33 ± 0.33^bcd^	5.47 ± 0.18^bc^
0.02	64.83 ± 3.08^ab^	32.83 ± 0.87^ab^	2.15 ± 0.11^ab^	6.5 ± 0.25^c^	30.58 ± 2.1^ab^	5.41 ± 0.16^bcd^	5.56 ± 0.20^abc^
0.03	66.16 ± 1.30^a^	36.5 ± 1.00^a^	2.15 ± 0.02^ab^	8.08 ± 0.71^bc^	33.16 ± 0.54^a^	9.33 ± 0.79^abcd^	6.60 ± 0.20^a^
0.04	65.33 ± 0.54^a^	36.66 ± 0.22^a^	2.21 ± 0.21^a^	10.41 ± 0.44^bc^	33.75 ± 3.38^a^	11.58 ± 0.98^ab^	6.56 ± 0.17^a^
0.05	63.16 ± 1.09^abc^	32.91 ± 0.91^ab^	1.60 ± 0.23^b^	12.41 ± 0.18^a^	34.25 ± 3.64^a^	12.83 ± 3.58^a^	6.50 ± 0.23^ab^
0.06	61.83 ± 3.00^abc^	31.41 ± 1.01^ab^	1.01 ± 0.05^c^	8.58 ± 2.04^c^	25.08 ± 0.22^abc^	10.5 ± 0.52^abc^	5.45 ± 0.23^bc^

^A^Each value is mean of triplicate and each replicate consisted of 3 plants. Mean ± SE followed by same letter in column of each treatment are not significantly different at p = 0.05 as determined by Tukey–Kramer HSD. ^B^Control with water, ^C^Bulk S (bulk saponin) dissolved in water, ^D^Bulk T (bulk thymol) dissolved in 1% DMSO (Dimethyl sulfoxide).

## Discussion

**Figure 8:**
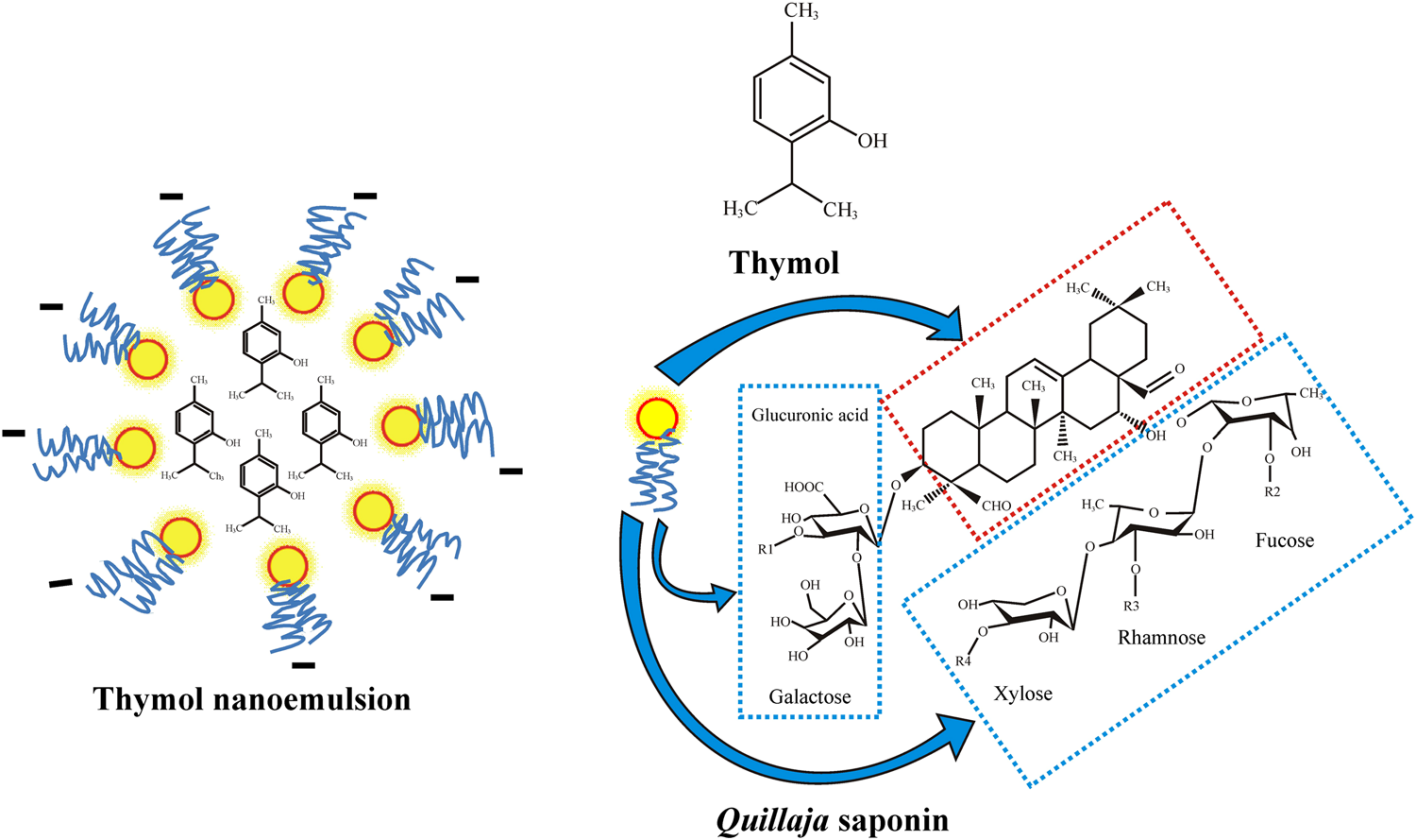
Hypothetical model of thymol nanoemulsion. Hydrophilic part of saponin consisted of glucuronic acid, galactose, xylose, rhamnose and fucose; R_1_-R_4_: H or alkyl group (blue rectangle) protruding to aqueous phase. Hydrophobic part of saponin that is triterpene sapogenin (red rectangle) remained toward thymol molecules. Carboxylate group (-COO^−^) of glucuronic acid mainly provides negative surface charge in thymol nanoemulsion. The structure of *Quillaja saponin* is adapted from reference (Wojciechowski *et al*.)^52^ with copyright permission from Elsevier.

In the present study, a stable, mono-dispersed, plant based thymol nanoemulsion was formulated by 50 min of sonication using thymol, saponin and water. The prepared thymol nanoemulsion demonstrated significant antibacterial, disease control and plant growth promoting activity. Fifty min of sonication delivered most stable nanoemulsion having z-average 274 ± 2 nm, PDI 0.1, and zeta-potential −31mV. Nanoemulsion synthesized by 10, 20, 30 and 40 min of sonication showed higher droplet diameters and higher PDI value as compared to 50 min of sonication. Increasing the sonication time from 10 to 50 min considerably decreased droplet diameters (458.0 to 274.7 nm) and PDI value (0.29 to 0.13) indicating that sonication time greatly influences the size, size distribution and subsequently the stability of nanoemulsion^29,39^. After 30 days of storage at room temperature, z-averages increased to 39.3, 35.5, 33.5, 24.6 and 1.82% in 10, 20, 30, 40 and 50 min sonicated nanoemulsions, respectively (Fig. 1b). Moreover, nanoemulsion prepared by less than 50 min of sonication, demonstrated phase separation through creaming after 20–30 days of storage. However, the intensity of creaming was lesser in 30 and 40 min sonicated thymol nanoemulsions as compared to 10 and 20 min sonication. The results strengthen the fact that higher sonication time feeds more kinetic energy to emulsions^40–42^ and facilitates the reduction of droplet size and narrows size distribution through more adsorption of surfactant on hydrophobic droplet surfaces^40,42^. Reduced droplets size and narrow size distribution prevents droplets growth by inhibiting coalescence, flocculation and Ostwald ripening thereby provides long-term stability^33,43^. Therefore, 50 min of sonication time was sufficient for achieving mono-dispersed and stable thymol nanoemulsion with no appreciable change in size, PDI and z-average (Table 1). Higher zeta-potential of nanoemulsion is an important characteristic of stability which contributed to higher electrostatic repulsion among droplets^38^. The negative zeta-potential (−31mV) of thymol nanoemulsion at pH 5.5 was mainly contributed by the carboxylate group (-COO^−^) of glucuronic acid of saponin^44,45^. Interestingly, nanoemulsions prepared by 10, 20, 30 and 40 min of sonication showed slightly higher zeta-potential as compared to 50 min sonicated thymol nanoemulsion (Fig. 1d). This might be implicited majorly by two facts, (a) lower sonication time was not enough for adsorption of sufficient saponin molecules on the droplet surfaces and thus, the formation of saponin micelles occurred (b) additionally, certain ionizable surface active impurities in saponin might have contributed to slightly higher charges on droplets^38,43^. These facts can further be comprehended by the appearance of creaming because of merging of thymol droplets due to insufficient cover by saponin surfactant. While, 50 min sonicated nanoemulsion was appreciably stable in variable pH and dilution due to substantially mono-dispersed, small sized and strong repulsion between thymol nano-droplets, adequately coated by saponin. Based on the study, a hypothetical model of thymol nanoemulsion was proposed, where hydrophilic part of saponin protrudes into aqueous phase and hydrophobic part remains towards thymol molecules (Fig. 8). Carboxylate group (-COO^−^) of glucuronic acid of hydrophilic moiety of saponin majorly contributed to negative surface charges (Fig. 8). Intense high pressure and cavitations in sonication process during emulsification might induce chemical deformation of oil phase^46^. Keeping this in view, FTIR spectra of bulk thymol and thymol nanoemulsion were studied for any chemical change in thymol and its interaction with saponin. Besides a broad peak at 3331 cm^−1^ which showed H-OH interaction in nanoemulsion, no specific peak was evident, thus specifying that thymol was chemically stable and simply entrapped within saponin^19,47,48^. TEM and Cryo-FESEM micrograph inveterated spherical shaped droplets of 80–150 and 90–180 nm diameters (Fig. 3a–c). The size measured by DLS on number distribution showed that 97.8% of droplets diameters were below 100 nm (Fig. S3; Table S3). *Quillaja* saponin is a mixture of different saponins^38^, hence, it is perceived that different surface active saponin micelles of above 100 nm size might be present in the emulsion which interact differently to thymol and due to that, z-average value of droplet diameter was on higher side (274 nm). Thymol nanoemulsions have so far been obtained mainly by synthetic and few natural surfactants^15,16,19,24,29,31–35^. Amongst the natural surfactants, *Quillaja* saponin is non-ionic, biodegradable, low oil soluble natural surfactant (Hydrophilic-lipophilic balance: 13) which can competently stabilize the nanoemulsion by imparting high interfacial charge and low interfacial tension in comparison to other surfactants^37,44,45,49^. Practically, surfactant concentration is kept high to achieve lower droplets size and higher stability of nanoemulsion^15^. However, in the present study we kept the saponin concentration comparatively low and achieved a substantially lower droplets size and remarkably stable nanoemulsion. Generally, higher concentration of surfactant may append toxicity and affect the biological activity of main component of nanoemulsion^31^. Therefore, nanoemulsion obtained in present study with low concentration of saponin can safely be used in crop and food and reduce the environmental burdens^36^.

Thymol nanoemulsion prepared by 50 min sonication imposed strong antibacterial activity against *X. axonopodis* pv. *glycine*. At the concentration of 0.01–0.06% (v/v), it significantly impeded bacterial growth as compared to control, bulk thymol and bulk saponin treatments (Fig. 5a,b) and distinctively, no bacterial colony was recorded in 0.02 to 0.06% (v/v) of nanoemulsions. Traditionally, thymol is known as a sturdy antimicrobial agent^11,13,14,50,51^. Earlier studies have proved that thymol nanoemulsions have more weighty effect on inhibition of bacteria growth as compared to less water soluble bulk thymol^16,19,34,35^. This could be comprehended by fact that uniformly dispersed nano-droplets of thymol nanoemulsion can easily penetrate and disrupt the microbial membrane^1,51^. In present study, significant antibacterial activity of thymol nanoemulsion was noticed even at a very low concentration (0.01%; v/v) which was irrefutably much better than previously synthesized nanoemulsions^19,32,35^. Another fact can not be ruled out that saponin might be contributing to antibacterial activity of thymol nanoemulsion, in which saponin may induce lysis of cell membranes through its lipophilic moiety^52^ although, bulk saponin and thymol did not express measurable antibacterial activity as compared to control. In spite of higher negative surface (−31mV zeta-potential) of thymol nanoemulsion, adhesion of droplets (specifically lipophilic thymol and lipophilic saponin moiety) to plant and bacterial surfaces could be explained by the chemical linkage rather than electrostatic interactions^16^. In view of its strong antibacterial activity against *X. axonopodis* pv. *glycine* in Petri plates experiments, we carried out pot experiments to study its further protective efficacy against bacterial pustule incidence in soybean. Control (water), bulk thymol and bulk saponin showed significantly higher disease incidence (74-68%), whereas a remarkable lower disease incidence (29-3.3%) was found in 0.04 to 0.06% of thymol nanoemulsion (Table 3). Pattern of lesion formation and its further amalgamation was visually distinguishable in control (water) and thymol nanoemulsion (Fig. 6). A significantly lower disease incidence and higher PEDC of thymol nanoemulsion in pot experiments could be explained by (a) the direct antibacterial activity of thymol nanoemulsion and (b) induction of plant defense reactions by eliciting the synthesis of phenolic compounds and peroxidase activity^53,54^. It is crucial to unravel the effect of thymol nanoemulsion on plant growth, so that an optimized dose could be figured out for effective control of disease with sustained plant growth. Therefore, plant growth characters were measured and corroborated with thymol nanoemulsion doses. A significant stimulatory effect on plant height, root length, root fresh weight, number of nodules/plant, weight/nodule, number of pods/plant and 100 seed weight was recorded in nanoemulsion treatments. At 0.06% nanoemulsion treatment, plant showed decreased values of various growth parameters as compared to 0.02 to 0.05% of nanoemulsion treatments (Table 4). The observed results confirmed the superiority of thymol nanoemulsion over bulk thymol for antibacterial and plant growth promotory activity. Information regarding the interaction of thymol with plant is rudimentary, yet it strengthens the assumption that thymol component of nanoemulsion plays an important role in amending the biochemical responses in plant through mobilization of reserved food and increases the activity of antioxidant enzymes^54^. More insights into the mechanisms of thymol’s interactions with plants might be beneficial for its future exploitation as a plant growth promoting agent. Hence, future research is under progress to deduce the effect of thymol nanoemulsion on seedling growth and its effect on antioxidant and plant defense enzymes activities. Furthermore, developed nanoemulsion is under investigation in field application for disease control and yield.

## Conclusions

A rapid, easy and reproducible method has been developed first time for the preparation of stable thymol nanoemulsion by sonication using natural saponin as surfactant. The synthesized thymol nanoemulsion exhibited strong antibacterial activity, significant disease control, plant growth promotory activity and remained stable for 3 months. In addition, oil phase of nanoemulsion could be used for solubilization of poorly soluble or water immiscible agrochemicals for efficient delivery and enhancing bioavailability. Hence, the results of present study also open up a new avenue for development of bio-based carrier for delivery of bioactive compounds/agrochemicals for efficient use in agriculture.

## Methods

### Materials

Thymol, (EOC, Mol. Wt. 50,000) and *Quillaja* saponin (Mol. Wt. 56,000) were procured from Sigma-Aldrich, St. Louis, MO, USA. Deionized water was obtained from a Milli-Q water purification system (Millipore Co., Bedford, MA, USA). Dimethyl sulfoxide (DMSO), Luria Bertani Broth and King’s medium B were purchased from HiMedia, India. Seeds of moderately susceptible soybean cultivar “*JS-*335” were purchased from certified seed supplier. Culture of *Xanthomonas axonopodis* pv. *glycine* was obtained from the Department of Plant Pathology, Rajasthan College of Agriculture, Maharana Pratap University of Agriculture and Technology, Udaipur, India.

### Preparation of nanoemulsion

Nanoemulsion was prepared by sonication method as described earlier with certain modifications^40,55^. In brief, on the basis of preliminary experiments, thymol and saponin were blended in the ratio of 6:1 (w/v) in deionized water through sonication (Q500 sonicator, Qsonica, USA). Probe sonicator (equipped with ½ inch probe with 12.7 mm tip) was used (500 watts, frequency of 20 kHz) with 5 second pulse on/off, 60% amplitude at room temperature (25 °C). Variable time duration (10–50 min) of sonication was applied to achieve stable mono-dispersed thymol nanoemulsion.

### Mean droplet diameter, polydispersity index (PDI) and zeta-potential measurements

Mean droplet diameters (z-averages), size distribution, polydispersity index (PDI) and zeta-potential of nanoemulsions were measured by dynamic light scattering on Zetasizer Nano ZS90,(Malvern, U.K.) at 25 °C at a scattering angle of 90° in triplicates. DLS analyses were performed up to 30 days at an interval of 5 days and finally after 3 months of storage at room temperature (25 °C). Data of droplet size distribution by number was also recorded in DLS analyses, as per requirement for interpretation of nanoemulsions. All the samples were diluted 100 times with deionized water for DLS analyses.

### Transmission electron microscopy (TEM), Cryogenic-field emission scanning electron microscopy (Cryo-FESEM) and fourier transform infrared (FTIR) analyses

To reveal the internal structure and physical size, one drop of nanoemulsion was positioned on copper grid (200 mesh), stained with 2% phosphotungstic acid and kept for drying^49^. TEM micrographs were acquired using a transmission electron microscope at accelerating voltage of 200 kv (Tecnai 20, Philips Electron Optics, Holland). To examine the external surface and physical size of nano-droplets, Cryo-FESEM (Jeol make JSM 7600 F with Cryo Unit, Quorum make PP3000T,UK) was performed. Emulsion droplets were freezed in liquid nitrogen at −196 °C. The samples were fractured and sublimed at −90 °C for 10 min and then sputtered for 30 seconds at 10 mA. Images of the samples were finally captured at −140 °C. FTIR was used to determine the interaction of thymol with saponin. For bulk thymol analysis, KBr pellet method was adopted at 1:99 ratio of sample and KBr powder^56^. Attenuated total reflection (ATR) unit was used for analysis of nanoemulsion^47^. Measurement was carried out from 600–4000 cm^−1^ wave numbers with 1 cm^−1^ resolution using FTIR spectrophotometer (Alpha, Bruker, Germany) equipped with an ATR cell.

### Creaming index (CI) measurements, effect of pH and dilution stability of nanoemulsion

To depict the intensity of phase separation, CI of nanoemulsion was measured after 3 months of storage using the following equation (1) and (2)^57^.

1 $$ \% \,{\rm{CI}}={{\rm{H}}}_{{\rm{S}}}/{{\rm{H}}}_{{\rm{T}}}\times 100$$

2 $${{\rm{H}}}_{{\rm{S}}}={{\rm{H}}}_{{\rm{T}}}-{{\rm{H}}}_{{\rm{C}}}$$

Where % CI is the creaming index, H_S_ represents the height of stable phase, H_C_ represents the height of creamy layer at the top of emulsion and H_T_ represents the total height of emulsion. To find out the effect of pH, freshly prepared thymol nanoemulsion was placed in 10 ml test tubes and different pH values (3–9) were adjusted using HCl and NaOH. Mean droplet size (z-averages), PDI and zeta-potential were measured by DLS. For dilution stability, fresh and pure nanoemulsion was added in to deionized water with gentle stirring to obtain 500 and 1000-folds diluted nanoemulsion. Mean droplet size (z-averages) and PDI values were measured just after dilution^58^.

### Antibacterial activity of nanoemulsion by growth kinetic analysis and CFU measurements

Antibacterial activity of the thymol nanoemulsion was investigated using growth inhibition studies^59^. In brief, colonies of *X. axonopodis* pv*. glycine* were inoculated into 50 ml Luria broth (LB) medium and cultured at 29 ± 1 °C for 48 h. Fifty µl of mother culture was added to tubes containing 5.0 ml LB medium supplemented with various concentrations (0.01, 0.02, 0.03, 0.04, 0.05 and 0.06%, v/v) of thymol nanoemulsion along with control (water), bulk thymol (0.01%, w/v) and bulk saponin (0.01%, w/v). Cultures were kept on rotary shaker incubator at 29 ± 1 °C at 200 rpm. The bacterial growth was monitored for 72 h by measuring optical density (O.D.) at 600 nm using spectrophotometer (UV-VS Spectrophotometer 118, Systronics). The number of viable cells was quantified by measuring colony forming units (CFU). For CFU measurement, 1 ml culture samples of different treatments from the stationary growth phase (determined in growth kinetic analysis) were taken and diluted to 10^6^ folds. Diluted samples (100 µl each) were spread on King’s medium B plates to obtain better discrete colonies. The plates were incubated at 29 ± 1 °C for 48 h. After incubation, the number of viable cells were quantified and compared to evaluate the antimicrobial property of treatments.

### Effect of nanoemulsion on bacterial pustule disease and plant growth

Pot experiments were performed in a net house under average 90% relative humidity (RH) and 25 °C average temperature. Soybean seeds were treated for 4 h with deionized water (control), bulk thymol (0.01% w/v), bulk saponin (0.01%, w/v) and thymol nanoemulsion (0.01, 0.02, 0.03, 0.04, 0.05 and 0.06%, v/v). The treated seeds were dried on filter paper and placed in earthen pots filled with surface soil collected from fallow land of the research field of Maharana Pratap University of Agriculture and Technology, Udaipur, India. Artificial inoculation of *X. axonopodis* pv*. glycine* were carried out after 35 days of sowing as per the standard method^60^. In brief, for the preparation of inoculum, the bacterial strain was cultured on King’s medium B plates at 29 °C ± 1 °C for 48 h. The bacterial culture was diluted with 10 mM MgCl_2_ to obtain 1 × 10^8^ CFU/ml at an optical density of 0.5 at 600 nm. Soybean plants were inoculated by spraying the bacterial suspension onto leaf surface using an atomizer. Foliar spray of thymol nanoemulsion (0.01, 0.02, 0.03, 0.04, 0.05 and 0.06%, v/v) along with control (water), bulk thymol (0.01%, w/v) and bulk saponin (0.01%, w/v) was applied after disease emergence until run-off using Knapsack battery sprayer (YS-095-2, Yes international, India). For bacterial pustule disease assessment, leaves from each replicate were selected for observation. Disease severity (DS) was evaluated on a scale of 0 to 5 (leaves with no visible symptoms = 0; few individual lesions = 1; many individual lesions = 2; small patches of coalesced lesions = 3; medium sized patches of coalesced lesions = 4; and large patches of coalesced lesions = 5)^61^. Further, DS and percent efficacy of disease control (PEDC) were calculated using the formula (3 and 4) given by Chester^62^ and Wheeler^63^.

3 $${\rm{DS}}={\rm{Sum}}\,{\rm{of}}\,{\rm{all}}\,{\rm{individual}}\,{\rm{disease}}\,\mathrm{rating}/\mathrm{total}\,{\rm{number}}\,{\rm{of}}\,{\rm{leaf}}\,{\rm{assessed}}\mbox{--}{\rm{maximum}}\,{\rm{rating}}\times 100$$

4 $${\rm{PEDC}}={\rm{Disease}}\,{\rm{severity}}\,{\rm{in}}\,{\rm{control}}\mbox{--}{\rm{disease}}\,{\rm{severity}}\,{\rm{in}}\,\mathrm{treatment}/\mathrm{disease}\,{\rm{severity}}\,{\rm{in}}\,{\rm{control}}\times 100$$

After physiological maturity, plant height, root length, root fresh weight, number of pods/plant and 100 seed weight were recorded. Number of nodules per plant and weight/nodule were measured after 45 days of sowing.

### Statistical analyses

Statistical analyses of the data was performed with JMP software version 12. The significant differences among treatment groups were determined using the Tukey-Kramer HSD at *p* = 0.05. All the experiments were performed in three replications (triplicates) and for pot experiment, each replication consisted of three plants.

## Electronic supplementary material

Supplementary Information
