# Supplementary material for: Thymol nanoemulsion exhibits potential antibacterial activity against bacterial pustule disease and growth promotory effect on soybean
Source: Sci Rep. 2018 Apr 27;8:6650. doi: 10.1038/s41598-018-24871-5 (PMC5923198; doi:10.1038/s41598-018-24871-5)
Supplement: Supplementary file 1 — Supplementary Information [file 41598_2018_24871_MOESM1_ESM.pdf]

Thymol nanoemulsion exhibits potential antibacterial activity against bacterial pustule disease and growth promotory effect on soybean

Sarita Kumari[†], R. V. Kumaraswamy[†], Ram Chandra Choudhary[†], S.S Sharma[¶], Ajay Pal[‡], Ramesh Raliya[§], Pratim Biswas[§] and Vinod Saharan*[†]

[†]Department of Molecular Biology and Biotechnology, Rajasthan College of Agriculture, MaharanaPratap University of Agriculture and Technology, Udaipur, Rajasthan 313 001, India

[¶]Department of Plant Pathology, Rajasthan College of Agriculture, Maharana Pratap University of Agriculture and Technology, Udaipur, Rajasthan 313 001, India

[‡]Department of Chemistry and Biochemistry, College of Basic Sciences and Humanities, Chaudhary Charan Singh Haryana Agricultural University, Hisar, Haryana 125 004, India

[§]Department of Energy, Environmental and Chemical Engineering, Washington University in St. Louis, MO 63130, USA

*Corresponding author

E-mail: vinodsaharan@gmail.com

Phone: +91-9461180586; Fax: +91-294-2420447

Supplementary information

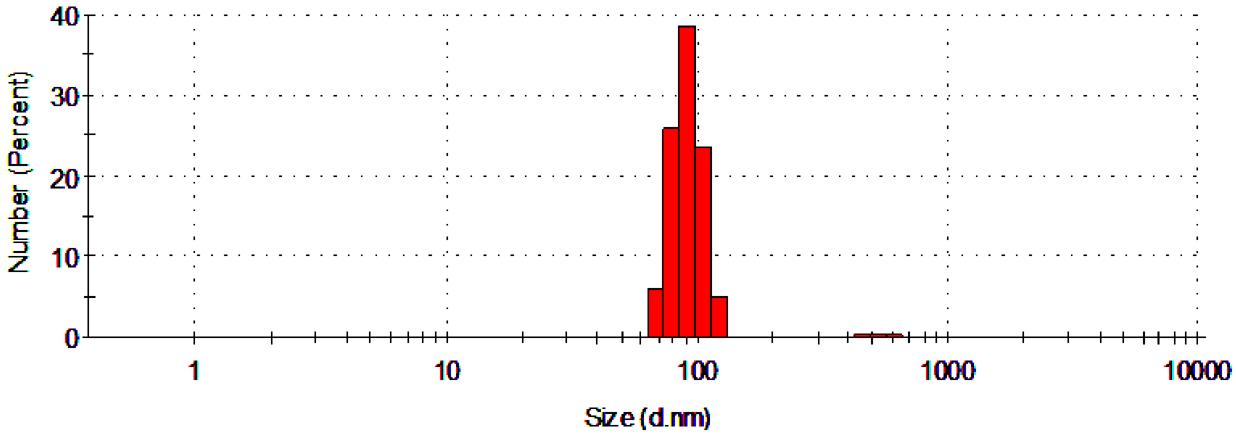

Figure S1. DLS analysis of 1000-folds diluted thymol nanoemulsion based on number distribution.

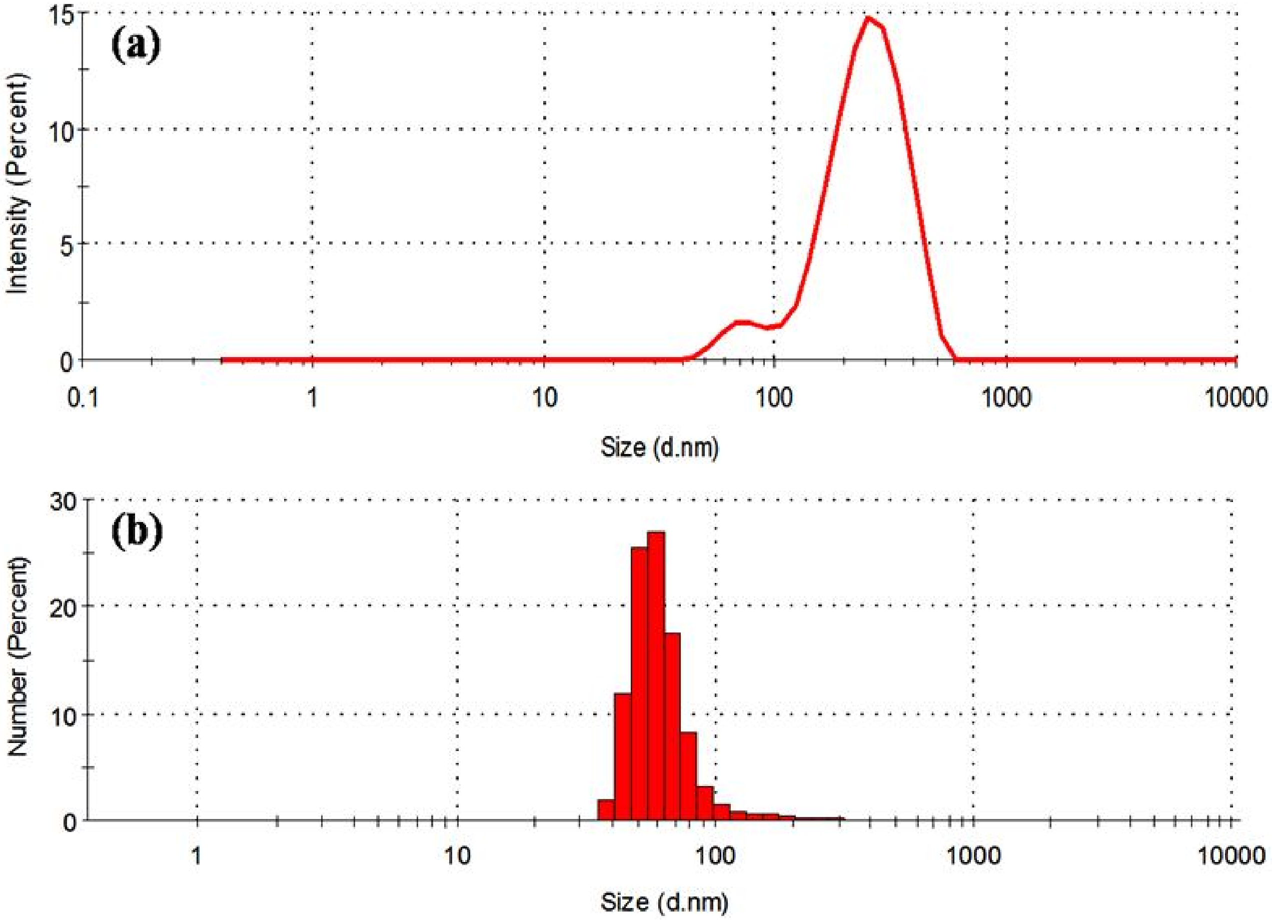

Figure S2. DLS analyses of 60 min sonicated thymol nanoemulsion (a) Bimodal size distribution and (b) number distribution.

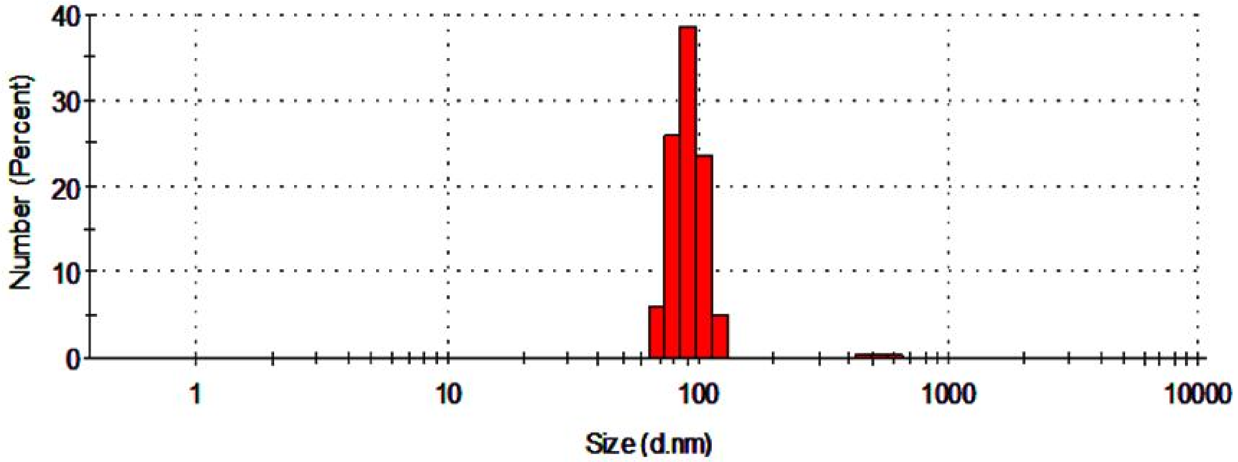

Figure S3. DLS analysis of 50 min sonicated thymol nanoemulsion based on number distribution.

Table S1. DLS analysis of 1000-folds diluted thymol nanoemulsion on % mean number versus size.

Size diameter (nm)	Mean number percent	Size diameter (nm)	Mean number percent	Size diameter (nm)	Mean number percent	Size diameter (nm)	Mean number percent
0.4	0	5.615	0	78.82	25.8	1106	0
0.4632	0	6.503	0	91.28	38.5	1281	0
0.5365	0	7.531	0	105.7	23.7	1484	0
0.6213	0	8.721	0	122.4	5	1718	0
0.7195	0	10.1	0	141.8	0	1990	0
0.8332	0	11.7	0	164.2	0	2305	0
0.9649	0	13.54	0	190.1	0	2669	0
1.117	0	15.69	0	220.2	0	3091	0
1.294	0	18.17	0	255	0	3580	0
1.499	0	21.04	0	295.3	0	4145	0
1.736	0	24.36	0	342	0	4801	0
2.01	0	28.21	0	396.1	0.1	5560	0
2.328	0	32.67	0	458.7	0.2	6439	0
2.696	0	37.84	0	531.2	0.3	7456	0
3.122	0	43.82	0	615.1	0.2	8635	0
3.615	0	50.75	0	712.4	0.1	1.00E+04	0
4.187	0	58.77	0	825	0		
4.849	0	68.06	6	955.4	0		

Table S2. DLS analysis of 60 min sonicated thymol nanoemulsion on % mean number versus size.

Size diameter (nm)	Mean number percent	Size diameter (nm)	Mean number percent	Size diameter (nm)	Mean number percent	Size diameter (nm)	Mean number percent
0.4	0	5.615	0	78.82	8.2	1106	0
0.4632	0	6.503	0	91.28	3.3	1281	0
0.5365	0	7.531	0	105.7	1.4	1484	0
0.6213	0	8.721	0	122.4	0.9	1718	0
0.7195	0	10.1	0	141.8	0.7	1990	0
0.8332	0	11.7	0	164.2	0.6	2305	0
0.9649	0	13.54	0	190.1	0.4	2669	0
1.117	0	15.69	0	220.2	0.3	3091	0
1.294	0	18.17	0	255	0.2	3580	0
1.499	0	21.04	0	295.3	0.2	4145	0
1.736	0	24.36	0	342	0.1	4801	0
2.01	0	28.21	0	396.1	0	5560	0
2.328	0	32.67	0	458.7	0	6439	0
2.696	0	37.84	1.9	531.2	0	7456	0
3.122	0	43.82	11.8	615.1	0	8635	0
3.615	0	50.75	25.4	712.4	0	1.00E+04	0
4.187	0	58.77	27	825	0		
4.849	0	68.06	17.4	955.4	0		

Table S3. DLS analysis of 50 min sonicated thymol nanoemulsion on % mean number versus size.

Size diameter (nm)	Mean number percent	Size diameter (nm)	Mean number percent	Size diameter (nm)	Mean number percent	Size diameter (nm)	Mean number percent
0.4	0	5.615	0	78.82	5.9	1106	0
0.4632	0	6.503	0	91.28	2.4	1281	0
0.5365	0	7.531	0	105.7	1	1484	0
0.6213	0	8.721	0	122.4	0.5	1718	0
0.7195	0	10.1	0	141.8	0.3	1990	0
0.8332	0	11.7	0	164.2	0.2	2305	0
0.9649	0	13.54	0	190.1	0.1	2669	0
1.117	0	15.69	0	220.2	0.1	3091	0
1.294	0	18.17	0	255	0.1	3580	0
1.499	0	21.04	0	295.3	0.1	4145	0
1.736	0	24.36	0	342	0	4801	0
2.01	0	28.21	0	396.1	0	5560	0
2.328	0	32.67	0	458.7	0	6439	0
2.696	0	37.84	6.1	531.2	0	7456	0
3.122	0	43.82	20.3	615.1	0	8635	0
3.615	0	50.75	27.9	712.4	0	1.00E+04	0
4.187	0	58.77	22.4	825	0		
4.849	0	68.06	12.8	955.4	0		
